# Long-Term Moderate Exercise Combined with Metformin Treatment Induces an Hormetic Response That Prevents Strength and Muscle Mass Loss in Old Female Wistar Rats

**DOI:** 10.1155/2019/3428543

**Published:** 2019-11-11

**Authors:** David Hernández-Álvarez, Beatriz Mena-Montes, Rafael Toledo-Pérez, Gibrán Pedraza-Vázquez, Stefanie Paola López-Cervantes, Alfredo Morales-Salazar, Edith Hernández-Cruz, Roberto Lazzarini-Lechuga, Roman Royer Vázquez-Cárdenas, Silvia Vilchis-DeLaRosa, Pedro Posadas-Rodríguez, Roberto Santín-Márquez, Oscar Rosas-Carrasco, Alejandra Ibañez-Contreras, Adriana Alarcón-Aguilar, Norma Edith López-Díazguerrero, Armando Luna-López, Mina Königsberg

**Affiliations:** ^1^Departamento de Ciencias de la Salud, Universidad Autónoma Metropolitana-Iztapalapa, Ciudad de México 09340, Mexico; ^2^Posgrado en Ciencias Biológicas, Universidad Autónoma Metropolitana, Ciudad de México 09340, Mexico; ^3^Instituto Nacional de Geriatría, SSA, Ciudad de México 10200, Mexico; ^4^Posgrado en Biología Experimental, Universidad Autónoma Metropolitana-Iztapalapa, Ciudad de México 09340, Mexico; ^5^Departamento de Biología de la Reproducción, Universidad Autónoma Metropolitana-Iztapalapa, Ciudad de México 09340, Mexico; ^6^APREXBIO S.A.S. de C.V., Mexico; ^7^Investigación Biomédica Aplicada (INBIOMA) S.A.S de C.V., Mexico

## Abstract

Sarcopenia is a syndrome characterized by a progressive and generalized skeletal muscle mass and strength loss, as well as a poor physical performance, which as strongly been associated with aging. Sedentary lifestyle in the elderly contributes to this condition; however, physical activity improves health, reducing morbidity and mortality. Recent studies have shown that metformin (MTF) can also prevent muscle damage promoting muscular performance. To date, there is great controversy if MTF treatment combined with exercise training improves or nullifies the benefits provided by physical activity. This study is aimed at evaluating the effect of long-term moderate exercise combined with MTF treatment on body composition, strength, redox state, and survival rate during the life of female Wistar rats. In this study, rats performed moderate exercise during 20 of their 24 months of life and were treated with MTF for one year or for 6 months, i.e., from 12 to 24 months old and 18 to 24 months old. The body composition (percentage of fat, bone, and lean mass) was determined using a dual-energy X-ray absorption scanner (DXA), and grip strength was determined using a dynamometer. Likewise, medial and tibial nerve somatosensory evoked potentials were evaluated and the redox state was measured by HPLC, calculating the GSH/GSSG ratio in the gastrocnemius muscle. Our results suggest- that the MTF administration, both in the sedentary and the exercise groups, might activate a mechanism that is directly related to the induction of the hormetic response through the redox state modulation. MTF treatment does not eliminate the beneficial effects of exercise throughout life, and although MTF does not increase muscle mass, it increases longevity.

## 1. Introduction

Aging is a gradual process characterized by a decrease in the overall homeostatic response, which leads to the accumulation of a wide variety of molecular and cellular damages associated with a gradual decline in physical and mental abilities [1, 2]. Likewise, aging is associated with profound changes in body composition; one of the main changes is the loss of skeletal muscle mass called sarcopenia [3, 4].

Sarcopenia is a syndrome that can lead to physical disability, walk disturbances, and falls that cause the loss of functional independence, increasing the health cost and eventually favors mortality risk [5, 6]. The etiology of sarcopenia is multifactorial, since it involves changes in both muscular and endocrine metabolism, nutritional aspects, as well as mitochondrial, oxidative stress, and genetic factors [7, 8]. The decrease of the physical activity and the functional limitations during aging favors a sedentary lifestyle reducing, in this way, the yield and muscular force, which predisposes the body to an increased risk of disability and disease [9]. Physical inactivity is clearly related to the loss of muscle mass and strength; conversely increased levels of physical activity can have protective effects [10]. Several longitudinal studies have shown that regular exercise can extend life expectancy and reduce the morbidity of the elderly [9]. The difference in prevalence of sarcopenia between men and women is somewhat controversial; some reports suggest that men have a higher incidence, while others propose the contrary [11–13]. However, it is known that in women, there is a special relationship between sarcopenia and osteoporosis. It has been reported that 68% of women diagnosed with sarcopenia have osteoporosis, while 20% of women aged diagnosed with osteoporosis have sarcopenia. Something similar happens in elderly men, but to a less extend [13, 14]. Therefore, in this study, we decided to use female rats as our experimental group.

Metformin (MTF) is a drug that belongs to the biguanide family; this medication has been highly prescribed for the type II diabetes treatment in the past 60 years [15]. Although its mechanism of action has not been fully elucidated, it is known that MTF inhibits hepatic gluconeogenesis by decreasing glucose plasma levels [16]. It has also been found that MTF inhibits mitochondrial respiratory chain complex I and generates low levels of oxidants, which induce the antioxidant response producing a hormetic effect. At the same time, reduced ATP levels activate AMPK, which activates diverse protective pathways [17]. It has been proposed that MTF prevents the damage caused by sedentary lifestyle by increasing the capacity of muscular performance [18], so MTF has been proposed as a geroprotective drug that can decrease the rate of aging [19, 20].

To date, there is great controversy if MTF treatment combined with exercise improves or nullifies the benefits provided by physical activity. There are reports in elderly people where the combined treatment eliminated the increase in mitochondrial respiration and insulin sensitivity given by exercise, without modifying the rate of protein synthesis, thereby abrogating the benefits of exercise on mitochondrial function [21]. Likewise, treatment with MTF significantly decreased exercise capacity in newly diagnosed patients with metabolic syndrome [22]. Other studies showed that MTF did not alter oxygen consumption or anaerobic threshold when administered to people who exercise, but it decreased the heart rate, exercise test duration, and respiratory rate ratio and increased rating of perceived exertion [23]. However, it has been reported that supplementing exercise with MTF (but not exercise alone), lowered proinsulin concentrations and increased insulin clearance in adults with prediabetes [24] suggesting a synergistic effect of both treatments. Similarly, combination treatment improved glucose tolerance and other parameters in women with polycystic ovary syndrome [25]. Eltonsy et al. also reported an improvement in glycated hemoglobin, functional capacity, lipid profile, quality of life, and body weight when using the combined treatment [26].

The problem with these great discrepancies is that the studies were performed for a short time in patients with or without different metabolic disorders; therefore, it is difficult to compare them. In this study, we used healthy Wistar female rats, which were treated and evaluated longitudinally throughout life. The rats performed moderate exercise for 20 of their 24 months of life (from 4 to 24 months old) and were administrated with MTF either for one year or for 6 months (from 12 to 24 months old and from 18 to 24 months old). Their size and weight were measured, as well as the percentage of fat, bone, and lean mass using a dual-energy X-ray absorption scanner (DXA). Grip strength and nerve somatosensory evoked potentials were also evaluated, as well as redox state by GSH/GSSG determination.

## 2. Materials and Methods

### 2.1. Chemicals

All chemicals and reagents were purchased from Sigma Chemical Co. (St. Louis, MO). The reagents obtained from other sources are detailed throughout the text.

### 2.2. Animals

One hundred sixty Wistar female rats (*Rattus norvegicus*) were used in this study. The animals were provided by the closed breeding colony from the Universidad Autónoma Metropolitana-Iztapalapa and were housed five per cage in polycarbonate cages, with reversed light-dark cycles and provided with standard commercial rat diet (Harlan 2018S, USA) and water *ad libitum*. All procedures with animals were strictly carried out according to the National Institutes of Health Guide for the Care and Use of Laboratory Animals and the Principles of the Mexican Official Ethics Standard 062-ZOO-1999 and the Standard for the disposal of biological waste (NOM-087-ECOL-1995). Chemical sedation was required for the application of the evoked potential tests and the DXA scan. The rats were anesthetized using an anesthetic mixture, which contained ketamine (100 mg/kg; Anesket® Pisa Farmacéutica Mexicana) and xylazine (5 mg/kg; Procin® Pisa Farmacéutica Mexicana) by intramuscular administration.

### 2.3. Experimental Groups

Rats were divided into 6 groups (*n* = 5): (1) sedentary rats (S), (2) sedentary rats that were treated with MTF from 12 months of age until their euthanasia at 24 months (one year of MTF treatment) (SM12), (3) sedentary rats that were treated with MTF from 18 months of age until their euthanasia at 24 months (six months of MTF treatment) (SM18), (4) rats that began their exercise routine from the age of 4 months and continued training until their euthanasia at 24 months (animals exercised for 20 months) (E); (5) rats that began their exercise routine from the age of 4 months and continued training until their euthanasia at 24 months and that were also treated with MTF for one year (from 12 to 24 months) (EM12), and rats that began their exercise routine at 4 months of age and continued training until their euthanasia at 24 months and that were also treated with MTF for six month (from 18 to 24 months) (EM18). MTF was daily administered orally at an already reported dose of 100 mg/kg body weight [27]. Since there are very few longitudinal studies to understand the effects of MTF at long-term treatment and, in particular, in combination with exercise training, our aim was to evaluate the long-term effect of that combined intervention and evaluate the differences when MTF treatment started at middle age or at old age. Our results related to the BMI and grip force showed that there are important changes at 12 and 18 months of age; consequently, those ages were considered to be middle age (12 months) and the beginning of old age (18 months). So MTF was administered to the sedentary and exercised rats starting at those ages.

### 2.4. Exercise Routine

Although there are different exercise training schemes, such as aerobic exercise, progressive resistance exercise, flexibility, and balance [28], our group has shown that moderate exercise throughout life is a good intervention to prevent sarcopenia in advanced ages [29]. This type of exercise simulates a light walk for half an hour and is suitable for old animals or elderly people where arduous exercise is difficult to perform. Consequently, here, the rats performed a mild exercise regimen, which started at 4 months of age and continued until their euthanasia at 24 months. The exercise routine was performed on the small animal treadmill (Panlab/Harvard Apparatus) at a controlled rate of 25 cm/s for 30 min a day, for 5 days a week during 20 months, as described by Mena-Montes and coworkers [29].

### 2.5. Morphometric and Biochemical Determinations

Animals were weighed and measured every 4 months in order to obtain the morphometric dimensions. For the biochemical determinations, the blood was collected during the animal euthanasia. Serum was obtained by centrifugation at 3500 rpm for 10 min. Subsequently, glucose, cholesterol, triglycerides, creatinine, GOT, GPT, GGT, and HDL were determined using the SPOTCHEM EZ SP-4430 chemistry analyzer.

### 2.6. Dual-Energy X-Ray Absorptiometry (DXA Scan)

The body composition was determined using the DXA scan (Serie Discovery QDR, Hologic® Discovery), at different ages (4, 8, 12, 18, 20, and 24 months of age). Prior to each evaluation, the equipment was calibrated with Hologic Rat Step Phantom P/N 010-0758. The body mass index, the total fat, the total bone, the total lean mass, and the gastrocnemius lean mass were obtained as described previously [29].

### 2.7. Histology

In order to evaluate muscle structure and integrity, hematoxylin and eosin (H/E) staining was performed in histological sections of the gastrocnemius muscle as described before [30].

### 2.8. Grip Strength

The grip strength was evaluated using a Rhino BAC-20 digital dynamometer (PKCh) with a measuring range between 0.1 N and 200 N (Newton). The dynamometer was vertically attached to a stainless steel grid, and the maximum gripping force was recorded at the time when a rat was released from the grid. The results are reported as N/kg-rat weight.

### 2.9. Evoked Potentials

In order to analyze another parameter related to movement functional deterioration, a noninvasive test that evaluates the neural sensory processing was performed. The evoked potentials (EP) were obtained in the six animal groups at 24 months of age, prior to their euthanasia, at APREXBIO's Neurophysiology Laboratory. EP were recorded in a NEURONICA 5® equipment (Neuronic®, La Habana, Cuba) using subcutaneous needle electrodes (Ambu®, Spain) located according with the 10/20 International System. The electrophysiological assays were registered employing <5 *Ω* impedances. Medial nerve somatosensory evoked potentials (MNSSEP) and tibial nerve somatosensory evoked potentials (TNSSEP) were obtained. To monitor MNSSEP, electrodes were placed at the 7° cervical vertebrae (C7) (-) and the contralateral superior somatosensory cortex (C3′ or C4′) (-) to the stimulated limb, referenced to Fz (+), and ground was placed at the mastoid. For TNSSEP registration, electrodes were placed at the following derivations: lumbar vertebrae (L5) (-) and the contralateral somatosensory cortex (C3′ or C4′) (-) to the stimulated limb, referenced to Fz (+), and ground was placed at the mastoid. The stimulus was a 100 *μ*s monophasic electric squared pulse, at 0.5-1.1 stimulus/s frequency, which was applied behind the medial malleolus. The filters used were from 5 to 2500 Hz, at a 1.5 mÅ intensity, and the response was replicated at least once to ensure the event reproducibility [31].

### 2.10. GSH/GSSG Determination by HPLC

The concentrations of GSH and GSSG in the gastrocnemius muscle, obtained from the six animal groups at 24 months of age, were determined as follows. Two hundred milligrams of the tissue was homogenized with 800 *μ*l of lysis buffer, 10% HCl, and 10 mM bathophenanthrolinedisulfonic acid (BPDS). Subsequently, the homogenate was centrifuged at 14000 rpm for 5 min at 4°C. Then, the supernatant was recovered and injected into the chromatographic system, which consisted of a binary 1525 pump (Waters, MA) connected to a Zorbax Eclipse XDB-C18 (Agilent, CA) reverse-phase column (250 × 4.6 mm i.d., particle size 5 *μ*m). The mobile phase consisted of acetonitrile (1%) and 20 mM potassium monobasic phosphate buffer, pH 7.0 (99%) at a 1 ml/min flow rate. GSH and GSSG were detected at 210 nm using a 2489 UV/Vis detector (Waters, MA). To determine GSH and GSSG concentration in the samples, standard curves were made using commercial standards at 5, 10, 25, 50, 100, 200, and 400 *μ*M for each reagent.

### 2.11. Statistical Analysis

Data are presented as a mean ± standard deviation of each animal group (*n* = 5). The population growth curves were established by polynomial correlations. The differences between the groups were determined by multivariate analysis (MANOVA). The differences in the redox state were determined by an ANOVA followed by a multiple comparison test of Tukey-Kramer. In all cases, the significance used is mentioned.

## 3. Results

### 3.1. Biochemical Determinations

The content of the transaminases Aspartate Aminotransferase (AST) and Alanine Amino Transaminase (ALT), as well as other parameters such as total cholesterol, HDL, triglycerides, creatinine, and fasting glucose levels, were evaluated at 24 months, when the animals were euthanized. The results obtained (Supplemental material, [Supplementary-material supplementary-material-1]) reveal that neither the treatment with MTF nor the exercise modified these parameters, which remained in the ranges reported by the Clinical Laboratory Parameters for Wistar rats [32].

Remarkably, only two parameters increased in comparison to the S group, the creatinine levels in exercised animals, and AST levels in all groups. However, neither of those parameters were out of the normal ranges reported [32].

### 3.2. Morphometric Analysis and Dual-Energy X-Ray Absorptiometry (DXA Scan)

**Figure 1:**
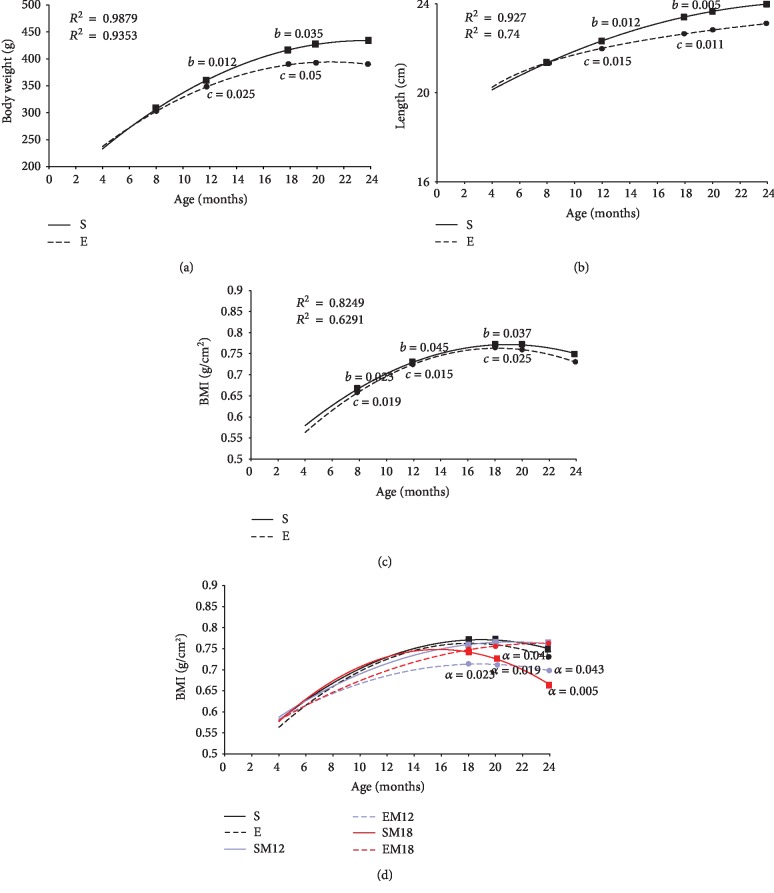
Body weight, length, and BMI. Wistar rat growth curves were performed by evaluating the body weight (a) and the nasorectal length (b). Using these data, the BMI for each group was obtained (c). Measurements were determined every 4 months (4, 8, 12, 18, 20, and 24 months) in sedentary (S) and exercised (E) rats. Each point in the curve represents the mean of all independent experiments carried out in each group of animals (*n* = 5); the growth curves were established using a polynomial adjustment (*R*^2^). Since the best parameter to define the different growth stages of Wistar rats is the BMI, this parameter was used to compare the different experimental groups (d). The experimental groups evaluated (S, E, SM12, EM12, SM18, and EM18) are described in detail in Materials and Methods. The differences between the experimental groups with respect to the sedentary group (S) are marked with the letter *a*. The exact probability value is indicated in the graph. The differences between ages were determined by comparing each age with its predecessor and are denoted as *b* for the sedentary rats and *c* for the exercised rats. The exact probability value is indicated in the graph. The comparisons were established by a multivariate test and corroborated with a Student *t*-test.

In order to better analyze our study groups and to compare the behavior of some important variables, we decided to transform the data as population groups that can be compared longitudinally and we made a polynomial correlation curve to describe the developmental stages of the rat. First, we performed a multivariate analysis of the morphometric variables body weight, nasorectal length, and body/mass index (BMI), comparing only the exercised rats vs. the sedentary rats at 8, 12, 18, 20, and 24 months of age (Figures 1(a)–1(c)). Then, a comparative analysis of the BMI including also the MTF treatment as a variable at 12, 18, 20, and 24 months of age was performed (Figure 1(d)).

In Figure 1(a), an increase in sedentary rats' body weight (BW) can be observed with respect to age with a correlation coefficient of *R*^2^ = 0.9879. Exercised rats showed a similar BW increase with a correlation coefficient of *R*^2^ = 0.9353. Both groups showed two stages where significant increases were be observed. The first one was between 8 and 12 months of age, where a significant BW increase was determined, 16.39% for sedentary rats (*p* = 0.012) and 15% for exercised rats (*p* = 0.025). The second staged was between 12 and 18 months, in which the sedentary animals showed a BW increase of 15.49% (*p* = 0.035) and the exercised animals of 13.04% (*p* = 0.05). The BM curves showed that sedentary rats were heavier than exercised rats.

Figure 1(b) illustrates the results obtained for the nasorectal length measurements. In both groups, the length increased with the age during the same periods of time where the weight augmented. From 8 to 12 months of age, the sedentary animals grew 5.18% (*p* = 0.012) and the exercised 4.24% (*p* = 0.015), while from 12 to 18 months of age, the sedentary rats increased their length 3.13% (*p* = 0.005) and the exercised rats 2.26% (*p* = 0.01). The nasorectal longitudinal curves showed correlation coefficients of *R*^2^ = 0.927 for the sedentary rats and *R*^2^ = 0.74 for the exercised rats.

Next, the BMI was evaluated. This parameter is commonly used to determine the nutritional status and development of organisms and provides indirect information about body composition.

The population analyzed showed very interesting results; both sedentary and exercised rats presented three stages in which the BMI changed (Figure 1(c)). The first one was between the 4 and 8 months of age, where an increase in BMI was observed, 8.5% for sedentary rats (*p* = 0.023) and 7.5% for exercised rats (*p* = 0.019). The second stage was between 8 and 12 months, where the BMI increased 16% for sedentary rats (*p* = 0.045) and 13% (*p* = 0.015) for those who exercised. In the third stage (from 18 to 24 months of age), the BMI increase for sedentary rats was 8% (*p* = 0.037) and 10% (*p* = 0.025) for exercised ones. The BMI was similar in both groups until 24 months of age. This parameter is relevant since it indicates the relationship between height and mass in the experimental groups in a more objective way, so it was the parameter that we used to compare the other experimental groups.

Due to the fact that important differences in the BMI were observed at 12 and 18 months of age and since those ages are considered to be middle age (12 months) and the beginning of old age (18 months), MTF was administered to the sedentary and exercised rats starting at 12 or 18 months of age. Sedentary animals that were treated with MTF starting at 12 months of age until their euthanasia at 24 months (one-year treatment) will be referred as SM12, while sedentary rats that were treated with MTF starting at 18 months of age until their euthanasia at 24 months (6-month treatment) will be referred as SM18. As regards to the exercised rats that were simultaneously treated with MTF, the ones that exercised for 20 months (from 4 to 24) and that took MTF from 12 to 24 months will be referred as EM12 and the ones that took MTF from 18 to 24 months and also exercised for 20 months will be called EM18.

As shown in Figure 1(d), our results indicate that the EM12 rats decreased their BMI by 9.97% at 18 months (*p* = 0.023), 9.93% at 20 months (*p* = 0.019), and 7.5% at 24 months (*p* = 0.043) when compared with the sedentary rats (S). Likewise, the SM18 rats decreased their BMI by 8% at 20 months (*p* = 0.045) and 13% at 24 months (*p* = 0.005) vs. S. The other experimental groups did not show significant differences in their BMI with respect to the sedentary group.

**Figure 2:**
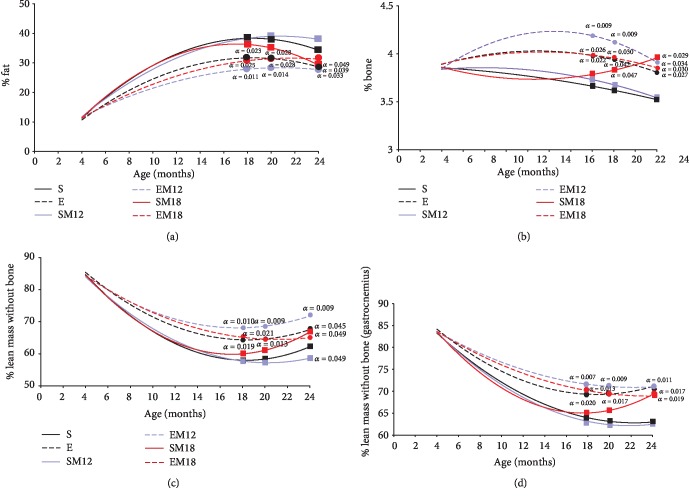
Body composition by dual-energy X-ray absorptiometry (DXA scan). The DXA scan was used to evaluate the percentage of the total body fat (a), bone (b), and whole *body* lean tissue *mass* or lean mass free of bone (LMWB) (c) as described in Materials and Methods. Gastrocnemius muscle LMWB was also determined (d). Each point in the curve represents the mean of all independent experiments carried out in each group of animals (*n* = 5); the growth curves were established using a polynomial adjustment (*R*^2^). Comparisons were made in the different ages and treatments with respect to the sedentary rats (*a*); the exact probability value is indicated in the graph. The comparisons were established by a multivariate test and corroborated with a Student *t*-test.

Next, we evaluated the body composition of all experimental groups using dual-energy X-ray absorptiometry (DXA scan). First, we determined the percentage of body fat. The results shown in Figure 2(a) indicate that the exercise groups exhibited 26.58% less body fat than the sedentary rats (*p* = 0.015) at the age of 12 months. This difference in weight is attributed exclusively to the exercise, because the MTF treatments had not yet started, since they started at 12 or at 18 months, respectively. The measurements of body fat percentage at 18 months of age revealed that the groups of rats that exercised still presented 21.05% less body fat than the sedentary group (*p* = 0.023 and *p* = 0.025). This is again attributed only to the exercise, since at the time of the 18-month DXA determination, the EM18 rats had not started yet with the metformin treatment. Interestingly, the EM12 rats given metformin for 6 months showed a 34.21% decrease in body fat with respect to sedentary rats (*p* = 0.011).

The body fat percentage at 20 months of age determined for the E and EM18 groups decreased 18.91% with respect to the S group (*p* = 0.028), while the EM12 group decreased 30.41% (*p* = 0.014). At 24 months, the fat percentage in the rats that exercised (E) decreased by 16.69% with respect to the S group (*p* = 0.039) and the EM12 group diminished by 16.78% (*p* = 0.033), while the SM18 group decreased 12.44% (*p* = 0.049). The other analyzed groups did not present significant differences, as can be seen in Figure 2(a).

The analysis of the curves of bone composition (Figure 2(b)) revealed an increase in 8.1% (*p* = 0.006) of bone in the E group and of 13.51% (*p* = 0.001) in the EM12 group with respect to the S group at 12 months of age. The determinations at 18 months indicated that the bone composition was higher in the E and EM18 groups (6.75%; *p* = 0.026 and *p* = 0.022) and in the EM12 group (12.16%; *p* = 0.009) with respect to the S group. At 20 months, the groups E, EM12, EM18, and SM18 showed an increase in bone percentage of 6.9, 11.55, 7.1, and 4.5%, respectively, with respect to the control (*p* = 0.042, *p* = 0.009, *p* = 0.030, and *p* = 0.047, respectively). Finally, the analysis of the composition of bone percentage at 24 months in the groups E, EM12, EM18, and SM18 showed an increase of 6.45, 7.5, 6.65, and 12.5%, respectively, with respect to the sedentary group (*p* = 0.027, *p* = 0.034, *p* = 0.030, and *p* = 0.029, respectively).

Whole *body* lean tissue *mass* (LMWB) or lean mass free of bone is an indirect way to evaluate muscle mass, so LMWB content was determined in order to understand the effect of exercise and MTF treatment on the muscle mass loss (Figure 2(c)). At 12 months of age, the exercised rats (E, EM12, and EM18) showed 12.24% more LMWB than the sedentary rats (*p* = 0.019). The determination at 18 months revealed that the E and EM18 groups had 12% and 11.95% more LMWB with respect to S group (*p* = 0.019 and 0.021), while EM12 showed an increase in LMWB of 19.65% with respect to the S group of the same age (*p* = 0.010). Groups E and EM18 maintained the difference of 12% in LMWB with respect to sedentary rats (*p* = 0.013 in both groups). A similar behavior was observed in the EM12 group with an increase of 19.60% with respect to the sedentary ones (*p* = 0.009). Finally, at 24 months, the E group had 8.02% more LMWB than the S group (*p* = 0.045), while the SM18 group presented 5.72% more LMWB (*p* = 0.049). The EM12 group showed the greatest difference at this age, 15.2% more than the S group (*p* = 0.009). Interestingly, the SM12 group showed 8.13% less LMWB with respect to the S group (*p* = 0.049). These results showed that the differences found in the BMI of the experimental groups were directly related to the body composition, where changes in the composition of fat, bone, and LMWB were observed.

In order to evaluate more specifically the LMWB composition, the gastrocnemius muscle mass was determined (Figure 2(d)). The gastrocnemius muscle mass composition was similar to that observed in the total body analysis. At 18 months, it was observed that the E group increased 10.31% LMWB compared to the S group (*p* = 0.020), the EM18 group showed 12.69% more LMWB than the S group (*p* = 0.013), and the EM12 rats showed 15.07% more LMWB (*p* = 0.007). At 20 months, the analysis of the LMWB composition revealed that the groups E and EM18 had 12.9% more proportion of LMWB than the S rats (*p* = 0.017). Lastly, the gastrocnemius muscle composition at 24 months revealed that the E, the EM18, and the SM18 groups presented 13% (*p* = 0.017), 16% (*p* = 0.017), and 12% (*p* = 0.019) more LMWB than the S group, respectively.

### 3.3. Grip Strength

**Figure 3:**
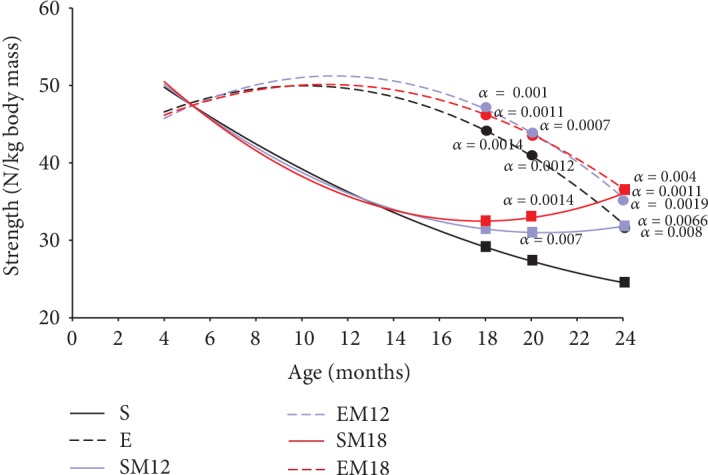
Grip strength. Grip strength was determined by dynamometry as described in Materials and Methods. The measurements are expressed in Newton per kilogram of rat weight. The data represented are the mean of 3 determinations in 5 different rats for each experimental group. The curve was adjusted by means of a polynomial regression. Comparisons were made in the different ages and treatments with respect to the sedentary rats (*a*); the exact probability value is indicated in the graph. The comparisons were established by a multivariate test and corroborated with a Student *t*-test.

To support the evaluation of the body composition and the gastrocnemius muscle, the strength was determined by dynamometry, as a parameter that helps to understand the skeletal muscle functionality (Figure 3). Our results showed that from 12 months the exercise groups (E, EM12, and EM18) had 34% more strength than the sedentary animals (*p* = 0.0001). This difference became greater at 18 months, since the animals in groups E, EM18, and EM12 showed more strength than those of group S. The E group increased 47.55% (*p* = 0.0014) and EM18 and EM12 by 59% (*p* = 0.0014, 0.0011, and 0.001, respectively). At 20 months, it can be observed that the MTF treatment in rats SM12 and SM18 increased their strength in 8.7 and 16.15%, respectively, with respect to the S group (*p* = 0.017 and 0.014), while the rats that exercised (E, EM12, and EM18) improved the strength in 44.63, 56.4, and 55.7%, respectively, with respect to the S group (*p* = 0.0012, 0.0007, and 0.0007, respectively). At 24 months, all the groups presented significant differences with respect to the S group and the increases in strength were 30 and 46.1% in the sedentary groups (SM12 and SM18) (*p* = 0.0066 and 0.0011), while the exercise groups (E, EM12, and EM18) increased their strength with respect to the S group in 29.78, 42, and 48.16% (*p* = 0.008, 0.0019, and 0.004), respectively.

### 3.4. Histology

**Figure 4:**
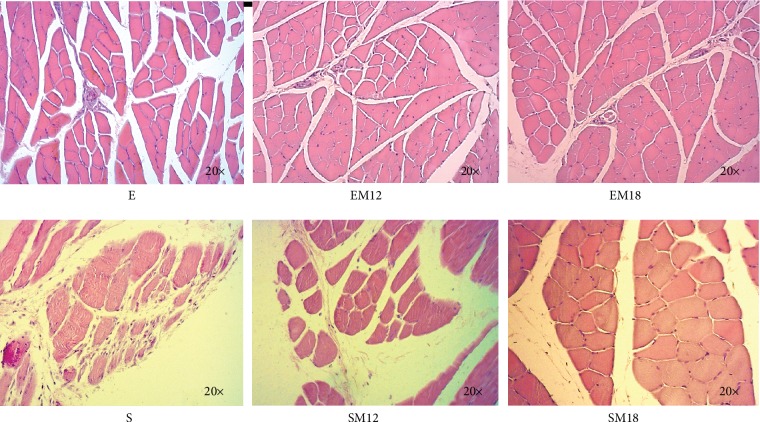
Histology, H&E. The images show the morphological analysis of the gastrocnemius muscle architecture in transversal sections. The presence of inflammatory infiltrates and the arrangement of muscle fibers in the different experimental groups were evaluated. The micrographs are representative images (20x amplification).

Figure 4 shows the H&E staining performed to the gastrocnemius muscle slices at 24 months of age, for the six animal groups. The sedentary group showed a total disorganization of the muscle fibers, since muscle fascicles are not observed. In addition, there is an adipose tissue accumulation and inflammatory infiltrates (Figure 4(d)). The groups that exercised (E, EM12, and EM18) presented an adequate arrangement of the muscle fibers, and the muscle fascicles and the myofibrils that compose them can still be observed. Interestingly, the accumulation of fatty tissue and inflammatory infiltrates can also be observed but in a lesser proportion than in the sedentary group (Figures 4(a)–4(c)). Regarding the structure of muscle tissue of sedentary groups treated with MTF (SM12 and SM18), it can be seen that muscle fascicles are larger compared to the tissues of the animals that performed exercise and also have less endomysium. Like the tissues of the other groups, they showed inflammatory infiltration and adipose tissue accumulation (Figures 4(e) and 4(f)).

### 3.5. Medial Nerve Somatosensory Evoked Potentials (MNSSEP) and Tibial Nerve Somatosensory Evoked Potentials (TNSSEP)

In order to evaluate another physiological characteristic of movement functional decline, we evaluated the MNSSEP and TNSSEP, which provide detailed information of the neural sensory processing time, by determining the electrophysiological responses when the nerves in the posterior and anterior extremities are stimulated. In this way, the neurophysiological assay complements the densitometric and grip strength studies.

**Figure 5:**
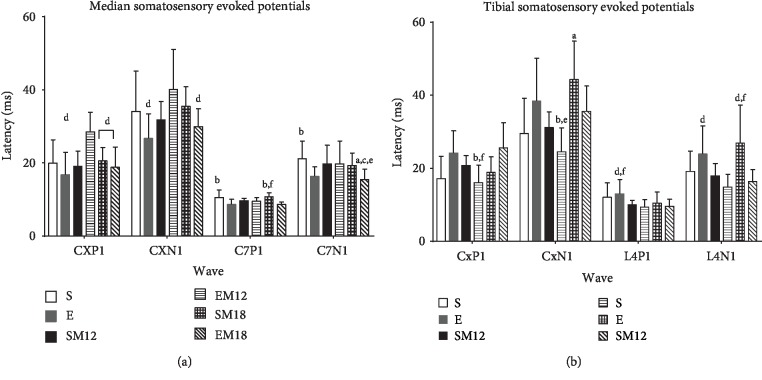
Somatosensory evoked potentials. Medial (a) and tibial (b) somatosensory evoked potentials were evaluated as described in Materials and Methods. Each bar represents the mean ± SD of all independent experiments carried out in each group of animals (*n* = 5). The Kruskal-Wallis test followed by Dunn's *post hoc* test were used to compare the data. The significance level is expressed as follows: (a) *p* < 0.05 vs. S, (b) *p* < 0.05 vs. E, (c) *p* < 0.05 vs. SM12, (d) *p* < 0.05 vs. EM12, (e) *p* < 0.05 vs. SM18, and (f) *p* < 0.05 vs. EM18.

Four components were obtained for the MNSSEP: CxP1, CxN1, C7P1, and C7N1. These waves reproduce the upper limb cortical pathway. As shown in Figure 5(a), all four latencies increased with age when compared to the young rats used as controls (4 months of age). This was expected since the nerve response becomes slower with age. However, no important differences were observed in the treated animals with respect to the S group in the upper limbs.

As regards to the lower limbs, the four components for the TNSSEP were also determined (CxP1, CxN1, L4P1, and L4N1) (Figure 5(b)). These waves reproduce the lower limb cortical pathway, and in this case, a more robust effect was observed in the improvement of the conduction velocity in the L4N1 wave in the rats that were treated with MTF and exercised, suggesting that these treatments might also impact, at least in part, the neural sensory processing.

### 3.6. GSH/GSSG Ratio

**Figure 6:**
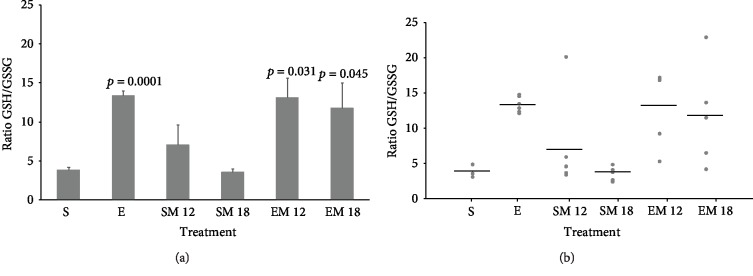
GSH/GSSG ratio. The concentrations of reduced glutathione (GSH) and oxidized glutathione (GSSG) were determined by HPLC using the gastrocnemius muscle obtained from animals of the 6 experimental groups at their euthanasia (24 months), as described in Materials and Methods. (a) The GSH/GSSG ratio is the mean ± SD (*n* = 5). (b) GSH/GSSG ratio for each of the analyzed animals. The horizontal line represents the average value in each group. To compare the groups, the ANOVA test was used followed by a Tukey-Kramer *post hoc* test. The exact probability value is indicated in each bar of the groups that presented differences.

To demonstrate that MTF, exercise, and the combined treatment participate in a mechanism that reduces oxidative stress associated to aging and sedentary lifestyle, we evaluated the GSH/GSSG ratio in muscle tissue, as an indicative of the tissue redox state. The results illustrated in Figure 6(a) show that the sedentary groups (S, SM12, and SM18) did not present significant differences in the GSH/GSSG ratio. However, the exercise groups (E, EM12, and EM18) presented increased GSH/GSSG ratio values.

It can be observed that the E group rats had a 3.5 times higher GSH/GSSG ratio compared with the S group rats (*p* = 0.0001), while group EM12 increased 3.45 times more its ratio (*p* = 0.031) and the EM18 group augmented the GSH/GSSG ration 3.08 times (*p* = 0.045). Interestingly, when the GSH/GSSG ratio was analyzed for each individual within the experimental groups (exercise, MTF treatments, and the combined treatment), it can be observed that 20% of the individuals in the group SM12 showed an increase in the GSH/GSSG ratio. Conversely, the same beneficial effect was not completely observed in the EM12 and EM18 groups, since only 60% and 40% of the population had higher levels of the mean value of the GSH/GSSG quotient (Figure 6(b)). The above suggests that MTF administration in combination with exercise might be a mechanism which involves an adaptive capacity to counteract stress which might be different for each individual in the population.

### 3.7. Kaplan-Meier Curves

**Figure 7:**
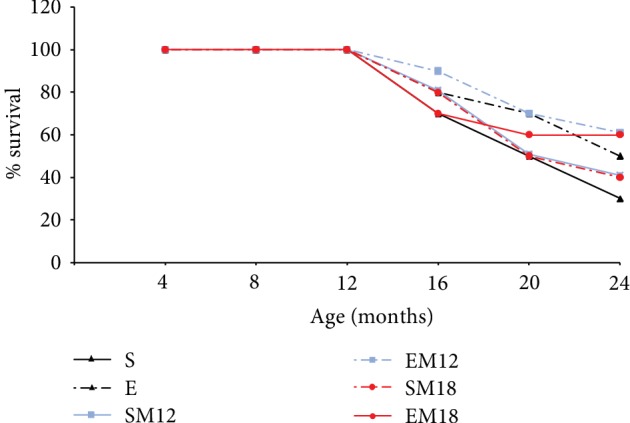
Kaplan-Meier survival curves. To evaluate the effect of exercise, MTF treatment, and the combination of both on animal survival, a Kaplan-Meier curve was performed. The total number of evaluated animals was 160.

To determine the effect of MTF, exercise, and the combined treatment on animal survival, we performed a Kaplan Meier curve. The results show that the lowest survival rate (up to 24 months) was found in the group of sedentary rats, with 30% survival, while groups of rats SM12 and SM18 had a 40% survival rate, which means an increase of 33% compared to the sedentary rats. With regard to the groups that exercised, it was observed that rats E and EM18 had a survival rate of 50%, which means an increase of 66% with respect to the sedentary group. The EM12 group had the highest percentage of survival, 60%, which represents an increase of 100% compared to the S rats (Figure 7). These results are very interesting since they show that treatment with MTF alone or in combination with exercise might increase survival.

### 3.8. Hormetic Response and Animal Survival

To determine the intensity and homogeneity of the hormetic response and their effect on survival, we established a model where we qualitatively related the redox state (GSH/GSSG) to the rats' survival at 24 months of age.

**Figure 8:**
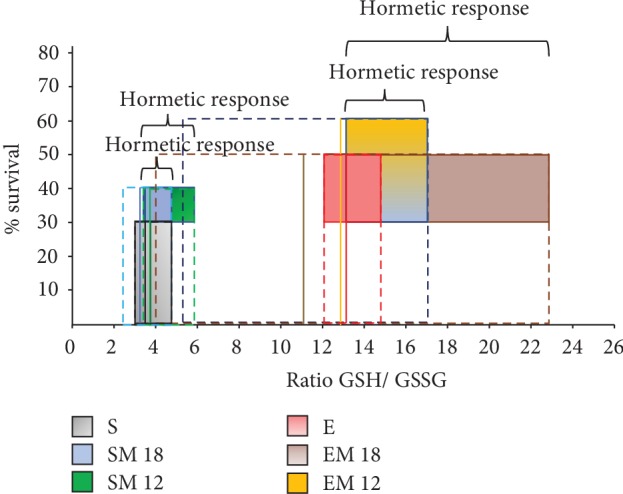
Hormetic response and animal survival. To relate animal survival with the GSH/GSSG ratio and to evaluate the effect that exercise, MTF treatment, and the combination of both might have on them, the hormetic response was analyzed. In order to do it, a “hormetic response” area was established using the minimum and maximum values obtained for the GSH/GSSG ratio with respect to the survival percentage at 24 months of age, in each group. The “hormetic response” area obtained for the sedentary group (S) was established as the reference standard. In the experimental groups, the dotted line represents the total area of the response induced with the different treatments. The shaded area represents the “hormetic response” induced with the treatments, that was determined as the area representing the values higher than the average of the GSH/GSSG ratio, both of the sedentary groups and of the exercise groups. The mean value of each treatment is represented by the continuous vertical line in each experimental group.

In Figure 8, the area of the “hormetic response” was delimited taking the minimum and the maximum values of the GSH/GSSG ratio and the survival percentage in each of the experimental groups (dotted lines). Arbitrarily, we established the area obtained for the sedentary rats as the reference area for survival vs. GSH/GSSG (i.e., SURV vs. GSH/GSSG). Subsequently, we determined the area of the “hormetic response” by plotting an area from the GSH/GSSG ratio mean value (continuous black line) of the sedentary rats and towards the maximum value of each of the treatments (shaded area). Our qualitative evaluation showed that the SURV vs. GSH/GSSG areas for the MTF treated groups (SM12 and SM18) were higher than those of the sedentary ones. Moreover, the SM12 group had a larger area than the SM18 group, suggesting that the redox state improves with the time of MTF administration, along with a homogeneity in the hormetic response, since the GSH/GSSG ratio interval was small. The same analysis of the SURV area vs. GSH/GSSG was made for the exercise groups. Group E was established as the reference area. The same thing was done as in the sedentary group. Our results revealed very interesting information, group EM18 presented a greater hormetic response area in relation to group E. However, the GSH/GSSG ratio interval was very large, suggesting that the hormetic response might be very heterogeneous and that the individuals in that group did not respond in the same way to the MTF treatment and exercise. With respect to the EM12 group, the results also showed a greater “hormetic response” area than the E group and a lower GSH/GSSG ratio interval than that the EM18 group, so this result suggests a more homogeneous adaptive response. In general, our analysis suggests that the time of MTF administration, both in the sedentary and the exercise groups, might activate a mechanism that is directly related to the induction of the hormetic response through the redox state modulation.

## 4. Discussion

A particular condition of frailty in the elderly is skeletal muscle atrophy or sarcopenia [33, 34]. The progressive loss of muscle mass produces a decline in both the force and the function of the tissue. The inability to move results in the deterioration of the quality of life, which is why sarcopenia has been considered as one of the illnesses that most increases fragility and therefore the disabling of the elderly.

For some decades, it has been known that exercise is the most effective intervention to delay the onset of sarcopenia in elderly people and murine models [35, 36]. However, although it is known that exercise increases both strength and muscle mass, the mechanisms by which this is carried out are not completely clear. It has been proposed that the anabolic response improves several signaling pathways, most notably the AMPK and AKT/mTOR pathways [37]. Exercise also increases mitochondrial biogenesis by activating PGC-1alpha and also reduces the secretion of proinflammatory cytokines [38]. It also decreases satellite cell death, preserving the myogenic capacity during aging [39], and it even increases the muscle antioxidant capacity [40].

Our results obtained with the exercise groups confirm that continuous exercise throughout life improved all the parameters evaluated and delayed sarcopenia to a great extent, since it prevented the loss of muscle and bone, avoided fat gain as well as inflammatory infiltration in muscle tissue, and protected against the disruption of fiber bundles. In addition, exercise prevented the loss of grip strength and showed lower state of oxidative stress (evaluated by the ratio of GSH/GSSG). The above could be related mainly to the loss of body fat, since it has been reported that adipose tissue generates a chronic, low-grade systemic proinflammatory state, with an increase in the levels of inflammatory cytokines such as TNF-*α*, IL-1*β*, and Il-6. These cytokines have been related to the muscle functional deterioration and to the strength loss, in a mechanism that involves a state of oxidative stress [41, 42]. However, the combined treatment with MTF for 12 or 6 months did not improve any of these parameters beyond what had already been preserved by the training alone (E groups), but interestingly it neither nullified nor eliminated them.

This is very important since recently two trials to evaluate MTF antiaging capacity with elderly people were approved, one with prediabetic [43] and the other one with healthy people [44, 45]; hence, knowing that, in an animal model, long-term MTF treatment used did not nullify the beneficial effect given by exercise supports the use of MTF for other antiaging effects.

With respect to the sedentary groups in which MTF was administered, interesting results were found. Administering MTF to adult rats for a period of 12 months did not induce BMI or body fat decrease, nor did it impedes or reduces the decrease in muscle or bone tissue. However, when MTF was administered to old rats for only 6 months at an old age (from 18 to 24 months of age), a significant decrease in BMI and fat percentage was found, as well as an increase in the percentage of muscle and bone tissues. Even though it has been reported that prolonged treatments with MTF decrease the body fat composition, this is rather controversial. An interesting meta-analysis using more than 50 studies in human adults was carried out, and only 10 of those studies strictly fulfilled the criteria for evaluation of body fat. Hence, only three of them concluded that MTF has moderate effects on the reduction of body mass [46].

A study in healthy adult men treated with two daily doses of MTF (850 mg) for 15 days showed no changes in body weight or body fat, although plasma glucose, insulin, and IGF-1 levels decreased [47]. In another study done in adult obese women without resistance to insulin, who were given 500 mg of MTF twice a day for three months, no differences were found in body weight, body fat, and blood pressure levels, neither did the glucose, insulin, and IGF-1 plasma levels decreased [48]. A recent meta-analysis [49] showed that MTF treatment in elderly humans moderately diminishes body weight, along with a significant decrease in serum cholesterol and LDL, which could suggest a reduction in the risk of major coronary events and of mortality in elderly diabetic populations.

In relation to the above, the differences observed in the responses between adult (12 months) and old (18 months) rats that were administered only with MTF could have several explanations; one of them might be related to the levels of sexual hormones that occur in these two different stages, estrogen and especially estradiol, a hormone that has been linked to the preservation of muscle and bone mass, as well as the accumulation of fat tissue [50]. Moreover, it has been reported that MTF administration reduces estradiol levels. Campagnoli and coworkers showed that 1500 mg of MTF daily for 9 months decreased estradiol levels in nondiabetic women, in a mechanism that involves the reduction of insulin levels [51]. Therefore, this effect could be related to the muscle and bone mass loss, observed in the treatment with MTF during 12 months in adult rats; interestingly, the MTF treatment for only 6 months at an elderly stage (from 18 to 24 months of age) might have a different mechanism of action. Thus, the hormonal levels might play an important role, which was beyond our study, but that should be analyzed in the future.

A very interesting observation was the adaptive response that each individual has against adverse changes in the environment, which can cause variations in gene expression that allows each one of them to survive, which is known as hormesis. This term, of pharmacological origin, refers to “the process by which an exposure to a low dose of a chemical agent or an environmental factor, which is harmful at high doses, induces an adaptive response and/or a beneficial effect on the cell or organism” [52–54]. In this sense, it has been reported that MTF can induce the hormetic response by ROS overproduction in a mechanism that involves the mitochondria and is known as “mitohormesis,” which has been related to the increase in longevity [55]. Moreover, it has been reported that this hormetic response is able to decrease the secretion of inflammatory cytokines such as TNF-*α*, IL-1*β*, and IL-6, because NF-*κ*B nuclear translocation and activation is abrogated [56]. In addition to MTF as a hormetic agent, our model has another stimulus capable of inducing the adaptive response, which is exercise. It has been reported that during moderate physical activity, muscle mitochondria increase their ROS production in order to modify redox state to promote an adaptive response rather an oxidative damage.

The benefits of this hormetic response include an increment in the antioxidant response to counteract environmental damage, as well as the ability to repair the damage generated by the inflammatory response, including low-grade chronic inflammation induced by adipose tissue [57]. It is important to note that during hormesis there are different response thresholds, which are in function of the stimulus intensity. In this model, we used two hormetic inducers, as well as their combination, to produce ROS and thereby modify the redox state and induce the adaptive response that has the effect of increasing survival. Interestingly, our results showed that some members of the groups EM12 and EM18 improved their GSH/GSSG ratio by 60 and 40%, respectively, increasing their reduced redox state to counteract the oxidative stress associated to aging.

This difference in the GSH/GSSG ratio could be linked to an adaptive hormetic process induced by MTF, which is supported by the increase in the population survival, which was 66% for the EM18 group and 100% for the EM12 group, when compared to sedentary rats (S) (Figure 7).

The increase in survival was also observed in the groups treated only with MTF, SM12 and SM18, where a 33% increase in survival was observed with respect to S group. A slight increase in the GSH/GSSG ratio was also found in these groups, which is why it is suggested that MTF is a hormetic agent capable of inducing such response. The differences in the hormetic response may be related to several factors linked to the individual's own capacities, including the ability to repair stress-induced damage, the ability to maintain homeostasis, the nutritional status, and the genetic background.

Finally, although there are some studies of diabetic neuropathies which suggested that MTF could recuperate nerve function [58, 59], our results of evoked potentials only showed an improvement in just one of the neural generators of the hind limbs in the animals treated with MTF, suggesting a very slight effect on the improvement of nervous conduction. In agreement with that, Caron et al. [60] reported that aging is associated to a decrease in muscle electrically induced fatigue, but that exercise training was unable to restore the responses. Since the evaluation with the EP only showed a slight effect, to elucidate this question, it would be desirable to conduct electromyography studies on the affected muscles such as gastrocnemius and quadriceps.

Some points that would be worth evaluating in the future to understand the interaction between exercise and MTF at the molecular level would be, first of all, the mitochondrial function, the ROS generation, and the ATP levels produced during these treatments, since the energy levels given by the ATP/AMP ratio might be activating the enzyme AMPK, which is an interesting link that is regulated by the MTF treatment [61] and by the exercise [62]. It would also be interesting to evaluate other metabolic functions that might be participating in the overall response such as hepatic and renal functions.

In conclusion, our work shows for the first time in a long-term longitudinal study, with healthy animals, that treatment with MTF does not eliminate the beneficial effects given by exercise throughout life. MTF might induce a positive hormetic response that protects the animals against damage and modifications in redox state, which in turn might improve lifespan, without eliminating other valuable effects such as the ones attained by exercise.

## Data Availability

The data used to support the findings of this study (morphometric and biochemical determinations, DXA-scan, histology, grip strength, evoked potentials, and GSH/GSSG) are included within the article.

## Supplementary Materials

Supplementary Materials Table 1: biochemical parameters.
