# Supplementary material for: Long-Term Moderate Exercise Combined with Metformin Treatment Induces an Hormetic Response That Prevents Strength and Muscle Mass Loss in Old Female Wistar Rats
Source: Oxid Med Cell Longev. 2019 Nov 11;2019:3428543. doi: 10.1155/2019/3428543 (PMC6877950; doi:10.1155/2019/3428543)
Supplement: Supplementary Materials — Table 1: biochemical parameters. [file 3428543.f1.pdf]

Supplemental material

Table 1. Biochemical Parameters.

The table shows the content of the biochemical parameters evaluated at 24 months, when the animals were euthanized. The experimental results are compared to the ranges reported by the

Test	S	SM 12-24	SM 18-24	E	EM 12-24	EM 18-24
Glucose (89-163 mg/dL)	97.33 ± 33.29	96.25 ± 6.02 a= 0.72	89.25 ± 20.56 a=0.70	92.8 ± 24.23 a=0.82	97.5 ± 6.85 a=0.72	70 ± 26.20 a=0.29
Cholesterol Total (23-97 mg/dL)	69.66 ± 22.14	84.25 ± 21.18 a= 0.42	101 ± 43.61 a=0.27	107.4 ± 26.99 a=0.10	113 ± 60.45 a=0.16	83.8 ± 27.99 a=0.46
HDL-c (32.8 mg/dL)	20.66 ± 7.37	21.5 ± 5 a= 0.85	22.75 ± 10.53 a=0.72	32 ± 9.02 a=0.11	32.5 ± 11.21 a=0.15	25.2 ± 9.83 a=0.52
Triglycerides (16-175 mg/dL)	98.66 ± 42.12	126.66 ± 37.28 a= 0.43	127.4 ± 87.80 a=0.55	117.6 ± 20.67 a=0.52	110.5 ± 43.74 a=0.73	163.2 ± 58.91 a=0.12
Creatinine (0.3-0.6 mg/dL)	0.2 ± 0	0.25 ± 0.057 a= 0.17	0.2 ± 0 a=1.000	0.62 ± 0.08 **a=0.0001**	0.525 ± 0.22 **a=0.037**	0.42 ± 0.31 a=0.24
AST (64-222 U/L)	55.66 ± 47.96	163.25 ± 42.07 **a= 0.03**	155 ± 23.84 **a=0.014**	183.2 ± 28.23 **a=0.011**	223.5 ± 48.67 **a=0.007**	187.75 ± 55.37 **a=0.02**
ALT (14-64 U/L)	15.33 ± 9.23	31 ± 2.94 **a= 0.03**	29.25 ± 8.95 a=0.07	26.6 ± 14.58 a=0.23	27.75 ± 10.62 a=0.16	25 ± 12.51 a=0.26

Clinical Laboratory Parameters for Wistar rats (26), named as test in the table.

Table 1: Biochemical Parameters
